# Interlayer Engineering of Layered VOPO_4_ Through Organic Intercalation for Enhanced Potassium Storage Kinetics

**DOI:** 10.3390/mi17050621

**Published:** 2026-05-19

**Authors:** Xuyun Peng, Shuang Fan, Jingfeng Tai, Jinqiu Zhang, Xinhua Qiu, Suliang Chen, Weihua Li, Yingmeng Zhang

**Affiliations:** 1 School of Sino-German Intelligent Manufacturing, Shenzhen City Polytechnic, Shenzhen 518100, China; 2 Yangtze Delta Region Institute (Huzhou), University of Electronic Science and Technology of China, Huzhou 313000, China

**Keywords:** potassium-ion battery, VOPO_4_, interlayer engineering, phenylamine intercalation, organic-inorganic hybrid

## Abstract

Nonaqueous potassium-ion batteries (KIBs) are emerging as promising next-generation energy storage systems owing to their abundant resources and high energy density. However, their large-scale application is hindered by structural degradation and sluggish kinetics resulting from the large ionic radius of K ions. Engineering electrode materials with open frameworks, such as two-dimensional (2D) layered structures, present an effective strategy to address these challenges by providing rapid ion diffusion pathways and robust host structures. Herein, a rational interlayer engineering strategy is developed by intercalating phenylamine derivatives with varying molecular sizes (*P*-butylaniline: PTA, *P*-Methylaniline: PMA, and phenylamine: PA) into layered 2D VOPO_4_ nanosheets. The intercalation of PANI derivatives progressively expands the interlayer spacing from 0.76 nm (pristine VOPO_4_) to 1.58, 1.85, and 2.09 nm, while maintaining the structural integrity of the layered framework. Notably, the regulated interlayer expansion (from 0.76 to 2.09 nm) not only provides enlarged diffusion pathways for rapid K^+^ ion intercalation/deintercalation kinetics, but also facilitates the formation of oxygen vacancies that may serve as additional active sites for potassium storage. By correlating the electrochemical performance with the modulated interlayer distances, it is established that a preferred spacing of 1.85 nm achieves the best synergy between fast kinetics, high capacity, and structural stability. As expected, the electrode with the optimal interlayer spacing (1.85 nm) exhibits superior potassium-ion storage performance, delivering a high reversible capacity of 333.2 mAh g^−1^ at 0.1 A g^−1^ over 100 cycles and exceptional rate capability with 205.7 mAh g^−1^ retained at 1 A g^−1^, as well as maintaining remarkable stability up to 600 cycles even at high rates. This work innovatively proposes phenylamine derivative-enabled interlayer regulation as a promising approach for designing high-performance VOPO_4_-based electrode materials.

## 1. Introduction

The scarcity and geographically uneven distribution of lithium resources pose significant challenges in meeting the rapidly growing demand for high energy density lithium-ion batteries (LIBs) [1,2,3,4]. Consequently, potassium-ion batteries (KIBs) have emerged as a compelling alternative for large-scale stationary energy storage devices, mainly attributed to the low cost and high natural abundance of potassium (2.47% in the Earth’s crust, compared to only 0.0065% for lithium), as well as their similar manufacturing processes and electrochemical reaction mechanisms to those of LIBs [5,6,7,8]. Furthermore, the K/K^+^ redox couple exhibits a low redox potential of −2.93 V vs. SHE, which is comparable to that of Li/Li^+^ (−3.04 V). This enables a high operating voltage coupled with high energy density in full cells [9,10,11,12,13]. Moreover, K^+^ ions exhibit a lower desolvation energy and a smaller Stokes radius (3.6 Å) compared to Li^+^ (4.8 Å) and Na^+^ (4.6 Å), which results in higher ion conductivity and enhanced ion mobility in the electrolyte [14,15,16,17,18]. Nevertheless, the sluggish redox kinetics and inevitable volume variation induced by the larger ionic radius of K^+^ ions lead to the limited capacity and inadequate rate capability, severely hindering its practical application [19,20,21]. Hence, the design and development of advanced electrode materials capable of enabling efficient and sustained ion transport remains a significant challenge for the practical deployment of KIBs.

To date, a wide range of materials have emerged as promising electrode candidates for KIBs, including carbonaceous substances, layered structure materials and organic compounds. Befitting from simple and efficient alkali metal ion insertion/extraction chemistry, 2D-layered structure materials have garnered significant attention as electrode candidates for KIBs [22,23,24,25]. Particularly, inorganic layered compounds such as oxyphosphates (MOPO_4_, M = V, Nb) with a typical 2D layered structure and rich intercalation chemistry have generated strong interest, owing to their high theoretical capacity, excellent cycle stability and intrinsic safety, all of which arise from the robust host framework [26,27,28]. For instance, the promise of these materials in alkali-ion batteries is evident from their performance in lithium systems. Wen et al. prepared a reduced graphene oxide (rGO)-supported niobium oxyphosphate (NbOPO_4_) nanosheet (NbOPO_4_/rGO) as a high-performance anode material for LIBs, delivering a high specific capacity of 502.5 mAh g^−1^ at 0.1 A g^−1^ after 800 cycles along with an outstanding rate capability of 308.4 mAh g^−1^ at 8 A g^−1^ [28].

However, these materials still suffer from the drawbacks of sluggish reaction kinetics and structural instability during charge/discharge cycles, which are largely attributed to electrode pulverization, low conductivity and limited interlayer spacing. To address these limitations, several strategies have been developed. For example, engineering the interlayer spacing of layered structures boosts the electrochemical performance by accommodating more K+ ions, lowering the intercalation barrier and enhancing ion diffusion kinetics [23]. For instance, Zhou et al. successfully intercalated interlayer water and lithium ions into KMnO_2_, obtaining increased interlayer spacing from 6.34 Å to 6.93 Å. As a result, the optimal K_0.4_Mn_0.9_Li_0.1_O_2_·0.33H_2_O exhibited a capacity retention of 84.04% (vs. 28.09% for the undoped sample) and a greatly enhanced rate capability [29]. Li et al. increased MoS_2_ interlayer spacing from 6.2 Å to 9.8 Å (≈60% expansion) through oxygen incorporation or NH_4_^+^ intercalation, achieving a large capacity of ~520 mAh·g^−1^ under 200 mA·g^−1^ and a good rate capability for potassium-ion batteries [30]. Xing et al. expanded the interlayer spacing of WSe_2_ by incorporating oleylamine as an additive between the layers of WSe_2_, followed by high-temperature pyrolysis. This approach produced a two-atom-thick WSe_2_/C ultrathin nanosheet with an enlarged interlayer distance (from 0.651 to 0.755 nm), which delivered a high specific capacity of 384 mAh g^−1^ after 200 cycles at 0.1 A g^−1^ and maintained superior cycling stability over 500 cycles in high rates [31].

To reveal the critical influence of interlayer spacing on intercalation behavior, the development of novel layered materials with tunable interlayer distances represents a highly attractive approach, which enables the integration of a high specific capacity, superior rate capability and robust cycling stability [32,33]. Vanadium-based oxyphosphates, particularly VOPO_4_·2H_2_O, stand out among layered candidates due to their high theoretical capacity and versatile intercalation chemistry. It is also essential to investigate the relationship between K^+^ storage behavior and interlayer spacing in layered VOPO_4_·2H_2_O to unlock its full potential. Furthermore, conductive polymers can be considered as ideal electrode additives, as they enhance electrical conductivity and suppress volume expansion, thereby improving electrochemical performance. Additionally, they offer advantages such as environmental stability and facile synthesis [22]. Based on these considerations, the integration of high-capacity layered VOPO_4_·2H_2_O with interlayer spacing regulation through a rational intercalation strategy, potentially combined with conductive polymer additives, represents a promising approach for boosting potassium storage performance.

Herein, we report an effective different molecular-sized phenylamine derivative intercalation strategy to simultaneously modulate the interlayer distance of layered VOPO_4_·2H_2_O (0.76 nm) to construct a novel oxyphosphate architecture with outstanding potassium storage performance. It is noteworthy that three PA derivatives (PA, PMA, PTA) intercalated with VOPO_4_ (labeled as VOPO_4_-PANI, VOPO_4_-PANI-CH_3_ and VOPO_4_-PANI-(CH_2_)_3_CH_3_) phases with progressively increased d-spacings (1.58, 1.85 and 2.09 nm) were respectively synthesized. The intercalation of PA, PMA, and PTA with different molecular sizes greatly regulated the interlayer spacing and provided more active sites to boost ion diffusion and accommodate the volume change during the potassiation/depotassiation process, meanwhile buffering large volume changes and stabilizing the host structure during potassium (de)intercalation. In this work, the dependence of the capacity and cycling stability on interlayer spacing is systematically investigated. The VOPO_4_-PANI-CH_3_ with interlayer spacing (1.85 nm phase) delivers a high discharge capacity of 333.2 mAh·g^−1^ at 0.1 A·g^−1^ and optimized cycling stability over 600 cycles with a capacity retention of ~205.7 mAh·g^−1^ at 1.0 A·g^−1^.

## 2. Materials and Methods

### 2.1. Fabrication of VOPO_4_·2H_2_O Bulk

The VOPO_4_·2H_2_O bulk was synthesized based on a hydrothermal process with some modifications. In detail, 4.8 g V_2_O_5_ and 26.6 mL H_3_PO_4_ were added into 115.4 mL of deionized water under stirring for 30 min at room temperature. Subsequently, 10 mL of HNO_3_ was added dropwise to the above solution to ensure the oxidation state of vanadium, with vigorous stirring for 10 min. The mixture was then sealed in a 150 mL Teflon-lined autoclave and heated at 110 °C for 16 h. The obtained yellow precipitate was obtained by centrifugation, washed with DI water and acetone three times, and collected at 60 °C from the vacuum oven.

### 2.2. Intercalation of P-Butylaniline (PTA), P-Methylaniline (PMA) and Phenylamine (PA) into the VOPO_4_·2H_2_O Interlayer Space

To introduce corresponding phenylamine derivatives into the interlayer spacing of VOPO_4_·2H_2_O, 300 mg of VOPO_4_·2H_2_O bulk was dispersed into 30 mL of isopropanol in a 50 mL three-necked flask with the dropwise addition of 0.5 mL PTA, PMA and PA, respectively. The mixture was heated at 60 °C under continuous magnetic stirring for 2 h to obtain the corresponding phenylamine derivatives intercalated with VOPO_4_·2H_2_O with different interlayer spacing (VOPO_4_-PANI, 1.58 nm, VOPO_4_-PANI-CH_3_, 1.85 nm and VOPO_4_-PANI-(CH_2_)_3_CH_3_, 2.09 nm), respectively. The final product was collected and washed with ethanol three times, and then vacuum-dried at 60 °C for 12 h.

### 2.3. Material Characterizations

X-ray diffraction patterns (XRD, Rigaku D/max 2500 pc, Rigaku Corporation, Tokyo, Japan; Cu Kα radiation: λ = 1.5406 Å), Fourier transform infrared spectra (FTIR, Nicolet NEXUS 670, Thermo Nicolet Corporation, Madison, WI, USA), and Raman spectra (Renishaw Raman microscope, Renishaw plc, Wotton-under-Edge, UK) were applied to detect the relevant phase structure. The surface chemical composition and elemental states of the sample were examined by an X-ray photoelectron spectroscope (XPS, Thermo Scientific K-Alpha+, London, UK). The surface morphologies and microstructure of prepared samples were obtained by scanning electron microscopy (SEM, JSM-7800F, JEOL Ltd., Tokyo, Japan) and Transmission Electron Microscope (TEM, FEI Company, Hillsboro, OR, USA). Thermogravimetric (TG) analysis (NETZSCH-STA409PC, NETZSCH-Gerätebau GmbH, Selb, Germany) was performed under a heating rate of 10 °C/min from room temperature to 600 °C. 

### 2.4. Electrochemical Measurements

The relevant electrochemical measurements were carried out in coin-type 2032 cells. The working electrodes were prepared by pasting a slurry of the active materials, Super P, and poly(vinyl difluoride) (PVDF) in an 8:1:1 weight ratio onto a Cu foil substrate. Finally, CR2032 coin cells were assembled using the prepared electrode, a potassium foil, and a glass fiber filter as the working electrolyte, respectively. The relevant electrolyte is 1.0 M potassium bis(fluorosulfonyl)imide (KFSI) dissolved in a mixed solvent of ethylene carbonate and diethyl carbonate (1:1 in volume). The average loading density of active materials for the KIBs was maintained at ~1.5 mg cm^−2^. Subsequently, cyclic voltammogram (CV) profiles in a potential window of 0.01–3.0 V and electrochemical impedance spectroscopy (EIS) measurements were conducted on a electrochemical workstation (CHI760E, CH instruments, Shanghai, China).

## 3. Results

**Figure 1:**
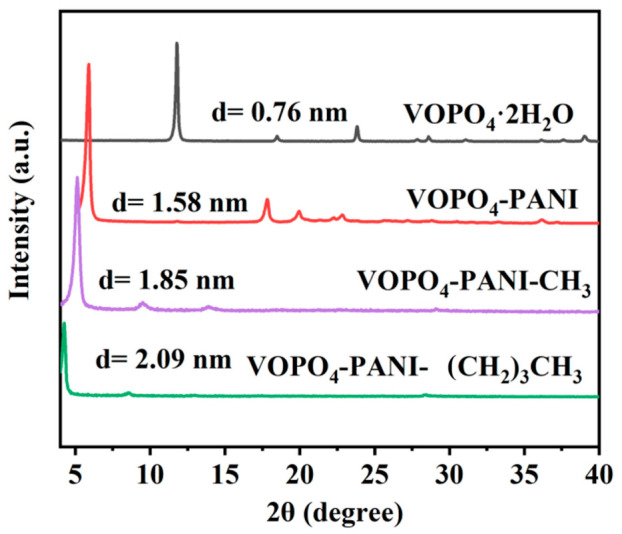
XRD patterns of the VOPO_4_-PANI, VOPO_4_-PANI-CH_3_, and VOPO_4_-PANI-(CH_2_)_3_CH_3_ hybrids, as well as VOPO_4_·2H_2_O.

As displayed in the X-ray powder diffraction (XRD) patterns in Figure 1, the diffraction patterns of the VOPO_4_-PANI, VOPO_4_-PANI-CH_3_, and VOPO_4_-PANI-(CH_2_)_3_CH_3_ exhibit a characteristic (001) reflection, confirming the retention of the lamellar structure after modification. All the diffraction peaks of the pristine VOPO_4_·2H_2_O are indexed to the tetragonal phase (JCPDS No. 84-0111) [33]. Upon intercalation of the PA derivatives, a distinct shift in the intense (001) peak toward a lower angle is observed, indicating a progressive expansion of the interlayer spacing. Specifically, the interlayer distance (d-spacing) increases from 0.76 nm for the pristine VOPO_4_·2H_2_O to 1.58 nm for VOPO_4_-PANI, 1.85 nm for VOPO_4_-PANI-CH_3_, and 2.09 nm for VOPO_4_-PANI-(CH_2_)_3_CH_3_. The interlayer distance enlargement is possibly attributed to the replacement of the lattice water molecules (weakly bounded) by the larger PA derivative species. It should be noted that the intercalated compounds (VOPO_4_-PANI, VOPO_4_-PANI-CH_3_ and VOPO_4_-PANI-(CH_2_)_3_CH_3_) display broad and less intense diffraction peaks compared to the pristine host, indicating a decrease in crystallinity upon intercalation.

The observed interlayer expansion correlates well with the molecular dimensions of the intercalated phenylamine derivatives. The molecular sizes of the three phenylamine monomer derivatives PA, PMA, and PTA are 0.64, 0.86, and 1.06 nm, respectively, while the corresponding interlayer spacings of these intercalated compounds expand by approximately 0.78, 1.07, and 1.31 nm relative to the pristine VOPO_4_·2H_2_O host (interlayer spacing: 0.76 nm). These expansions are consistent with the insertion of phenylamine monomer derivatives of varying molecular sizes into the interlayer space, as the increase in d-spacing follows the trend of the size of the guest species [34,35]. The slightly larger expansion compared to the molecular size may be attributed to the orientation of the inserted molecules (e.g., perpendicular vs. parallel arrangement within the interlayer gallery) or the co-intercalation of solvent molecules. These results confirm the successful intercalation of the phenylamine derivatives while maintaining the structural integrity of the VOPO_4_ host lattice. Furthermore, the decreased crystallinity observed in the intercalated composites, evidenced by broader and less intense diffraction peaks, suggests the formation of more grain boundaries and defects, which may provide additional active sites for potassium storage.

**Figure 2:**
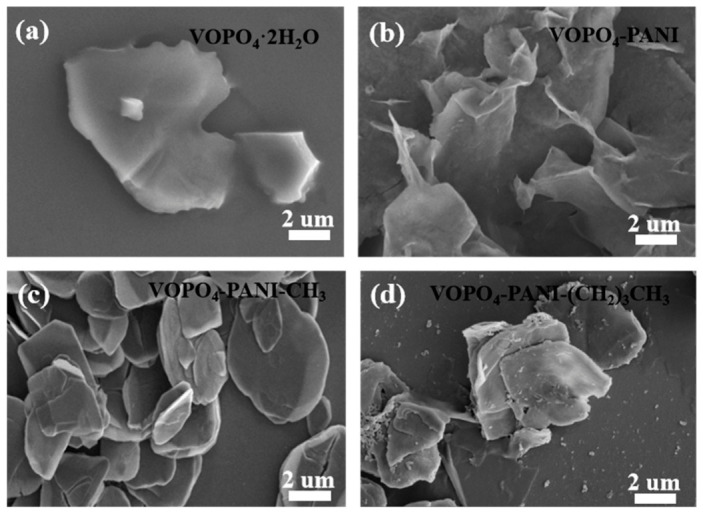
SEM images of (**a**) VOPO_4_·2H_2_O, and (**b**) the VOPO_4_-PANI, (**c**) VOPO_4_-PANI-CH_3_ and (**d**) VOPO_4_-PANI-(CH_2_)_3_CH_3_ hybrids.

**Figure 3:**
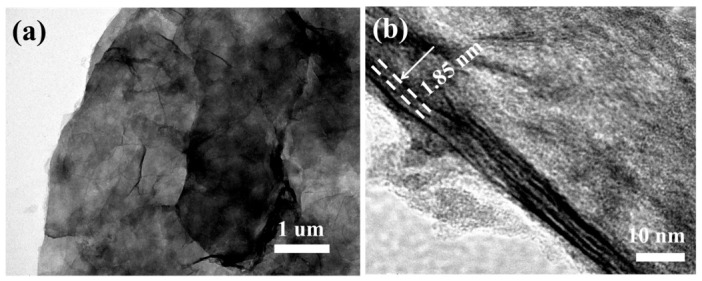
(**a**) TEM, and (**b**) HRTEM images of VOPO_4_-PANI-CH_3_ hybrids.

The morphology of the as-prepared materials was characterized by scanning electron microscopy (SEM). As shown in Figure 2, all composite samples exhibitde a typical flake-like structure, with lateral dimensions of the flakes ranging from 3 to 5 μm. A comparative analysis of the microstructures (Figure 2a–d) reveals that increasing the molecular size of the intercalated phenylamine derivatives leads to a progressive reduction in the thickness of the flake structure, which can be rationalized by the weakening of interlayer van der Waals interactions upon organic molecule insertion. The intercalated PANI derivatives effectively force apart the adjacent VOPO_4_ layers along the c-axis, thereby suppressing preferential growth in the vertical direction during synthesis. This controllable structural evolution is expected to be advantageous for potassium-ion storage, as thinner flakes can effectively shorten the ion diffusion pathways of K^+^ ions and provide a larger electrode/electrolyte contact area, thereby facilitating ion transport kinetics and potentially enhancing the overall capacity [36]. The transmission electron microscopy (TEM) images shown in Figure 3a exhibit the typical flake-like structure of the VOPO_4_-PANI-CH_3_ hybrids. Consistent with the XRD result, the high-resolution TEM (HRTEM) image in Figure 3b clearly displays a distinct interlayer spacing of ~1.85 nm [37].

**Figure 4:**
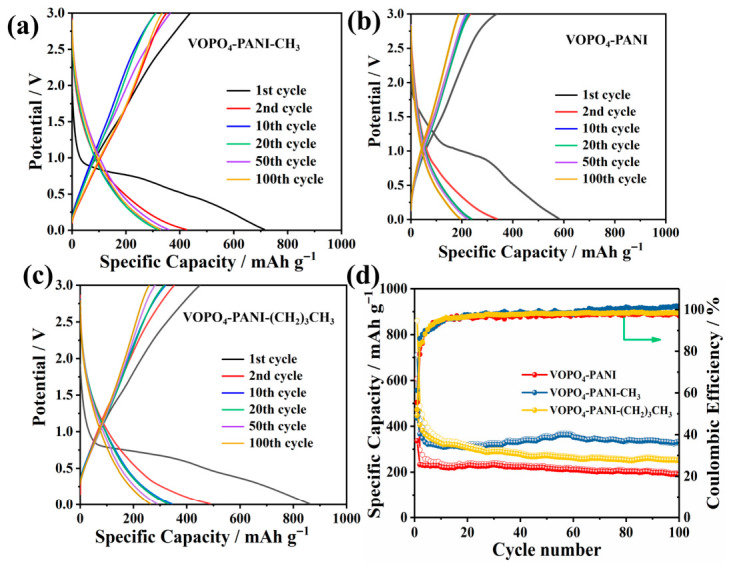
(**a**) Cycling performance of the VOPO_4_-PANI, VOPO_4_-PANI-CH_3_ and VOPO_4_-PANI-(CH_2_)_3_CH_3_ electrodes at 0.1 A g^−1^. Galvanostatic charge/discharge curves of the (**b**) VOPO_4_-PANI, (**c**) VOPO_4_-PANI-CH_3_ and (**d**) VOPO_4_-PANI-(CH_2_)_3_CH_3_ electrode at 0.1 A g^−1^.

**Figure 5:**
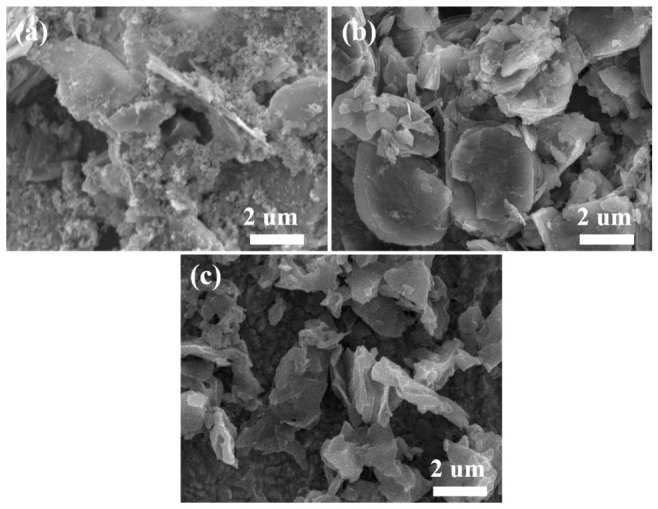
The SEM images at 10 cycles of (**a**) the VOPO_4_-PANI, (**b**) VOPO_4_-PANI-CH_3_ and (**c**) VOPO_4_-PANI-(CH_2_)_3_CH_3_ hybrids.

The potassium storage performance of VOPO4-PANI, VOPO_4_-PANI-CH_3_ and VOPO_4_-PANI-(CH_2_)_3_CH_3_ composites as anode materials for KIBs were systematically investigated (Figure 4). At a current density of 100 mA g^−1^, the VOPO_4_-PANI-CH_3_ electrode delivers an initial discharge and charge capacity of 711 and 435 mAh g^−1^, respectively (Figure 4a). The initial CE is 61%, which is mainly related to the irreversible electrochemical reaction, the decomposition of organic electrolyte, and the formation of the solid electrolyte interphase (SEI) layer [38]. Subsequently, the Coulombic efficiency gradually increases to 95% after 10 cycles and stabilizes thereafter. After 100 cycles, a reversible capacity of 333.2 mAh g^−1^ is retained for the VOPO_4_-PANI-CH_3_ electrode, indicating good cycling stability. In contrast, the electrochemical behavior of the other two composites strongly depends on their interlayer spacing. As shown in Figure 3b, the VOPO_4_-PANI electrode (Figure 4b), with its relatively smaller interlayer spacing (1.58 nm), exhibits a limited potassium storage capacity of only 190.4 mAh g^−1^ after 100 cycles at 0.1 A g^−1^, likely due to constrained ion diffusion pathways and insufficient space to accommodate the large K^+^ ions. Although the VOPO_4_-PANI-(CH_2_)_3_CH_3_ electrode (Figure 4c), featuring the largest interlayer spacing (2.09 nm), shows a higher initial specific capacity, it suffers from rapid capacity fading upon prolonged cycling, retaining only 291.5 mAh g^−1^ after 100 cycles (capacity retention of 58.3%). This degradation is likely attributable to structural collapse during repeated K^+^ extraction/insertion, as the excessively expanded interlayer gallery may compromise the mechanical integrity of the host framework, leading to electrode pulverization upon prolonged cycling (Figure 4d). After cycling, the VOPO_4_-PANI-(CH_2_)_3_CH_3_ composite shows severe morphological destruction, including cracked and exfoliated nanosheets (Figure 5c), whereas the other composites (VOPO_4_–PANI (Figure 5a) and VOPO_4_-PANI-CH_3_ (Figure 5b)) maintain the integrity of their flake-like structures. This observation confirms that excessive interlayer expansion weakens structural stability during repeated K^+^ intercalation/deintercalation, leading to capacity decay. In contrast, the moderately expanded samples exhibit well-preserved morphologies, supporting their superior cycling performance. Among the three electrodes, the VOPO_4_-PANI-CH_3_ electrode exhibits the most balanced electrochemical performance [37]. These results suggest the critical role of moderate interlayer expansion in simultaneously enabling facile ion transport and maintaining structural robustness for balancing high capacity with cycling stability [38,39,40,41,42].

**Figure 6:**
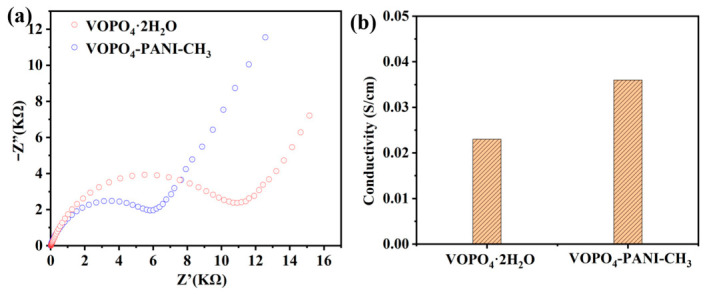
The Nyquist plots (**a**) and electrical conductivity (**b**) of pristine VOPO_4_·2H_2_O and VOPO_4_-PANI-CH_3_ electrodes.

Electrochemical impedance spectroscopy (EIS) was conducted to evaluate the charge transfer kinetics. The Nyquist plots (Figure 6a) reveal that the VOPO_4_-PANI-CH_3_ electrode has a much smaller charge transfer resistance (5.7 KΩ) compared to that of the pristine VOPO_4_·2H_2_O electrode (10.2 KΩ), indicating enhanced interfacial charge transport [43]. Four-point probe measurements were used to evaluate the electrical conductivity of the as-prepared pristine VOPO_4_·2H_2_O and VOPO_4_-PANI-CH_3_ electrodes, as presented in Figure 6b. The pristine VOPO_4_ exhibits an electrical conductivity of 0.023 S·cm^−1^, while the VOPO_4_-PANI-CH_3_ composite shows a significantly higher conductivity of 0.036 S·cm^−1^, confirming that the intercalation of PANI-CH_3_ effectively improves the electronic transport properties. These findings underscore that PANI-CH_3_ incorporation beneficially enhances charge-transfer kinetics at both the electrolyte and electrode interfaces for KIBs [44].

**Figure 7:**
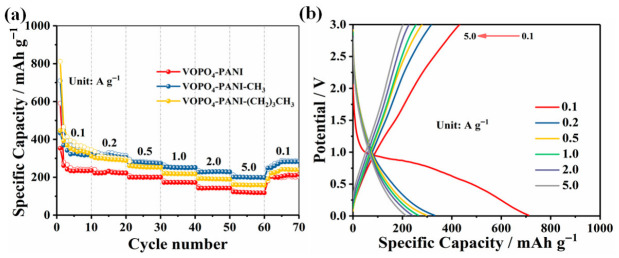
(**a**) The rate performance of the VOPO_4_-PANI, VOPO_4_-PANI-CH_3_ and VOPO_4_-PANI-(CH_2_)_3_CH_3_ electrodes at different current densities, and (**b**) the corresponding charge/discharge curves of the VOPO_4_-PANI-CH_3_ electrode at various current densities.

The remarkable capacity recovery to 298.9 mAh g^−1^ upon returning to 0.1 A g^−1^ demonstrates excellent electrochemical reversibility. The rate performance of VOPO_4_ and VOPO4-PANI-CH_3_ electrodes was compared at various current densities ranging from 0.1 to 5 A g^−1^, as shown in Figure 7. The VOPO_4_-PANI-CH_3_ electrode exhibits superior rate capability, with average specific capacities of 420.5, 320.1, 296.0, 275.3, 205.7 and 198.8 mAh g^−1^ at current densities of 0.1, 0.2, 0.5, 1, 2 and 5.0 A g^−1^, respectively (Figure 4a). Remarkably, when the current density was returned to 0.1 A g^−1^, a specific capacity of 298.9 mAh g^−1^ was recovered, indicating excellent electrochemical reversibility and structural resilience (Figure 7a). Furthermore, even at high current densities, the VOPO_4_-PANI-CH_3_ electrode retains distinct voltage plateaus (Figure 7b), indicating that the expanded interlayer spacing effectively buffers the mechanical strain associated with rapid K^+^ insertion/extraction at high rates. Among the electrodes tested, the VOPO_4_-PANI-CH_3_ composite exhibited the best electrochemical performance.

**Figure 8:**
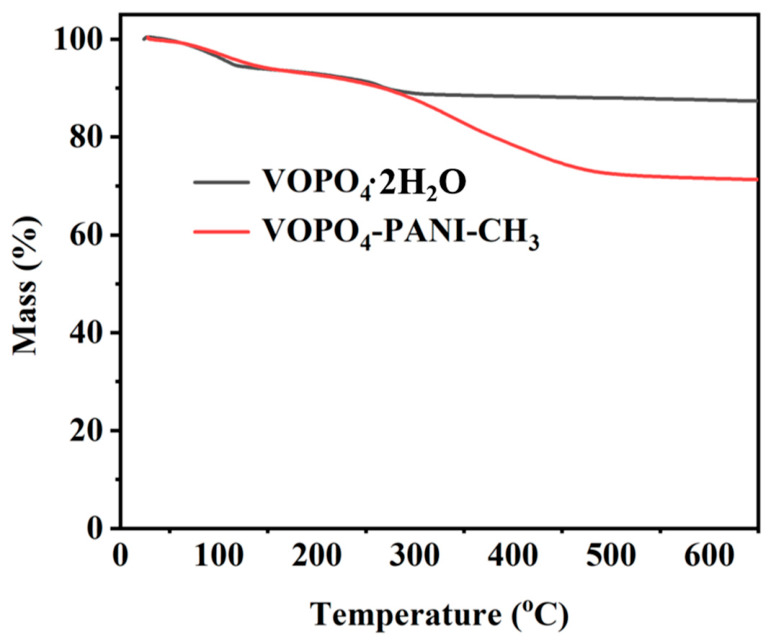
Thermogravimetric analysis (TGA) of the VOPO_4_·2H_2_O and VOPO_4_-PANI-CH_3_ hybrids.

To elucidate the structural and chemical origins of the enhanced electrochemical performance observed in the VOPO_4_-PANI-CH_3_ composite, thermogravimetric analysis (TGA), Fourier transform infrared (FT-IR) spectroscopy, and X-ray photoelectron spectroscopy (XPS) were conducted. TGA depicts two distinct weight loss stages for VOPO4-PANI-CH_3_ (Figure 8). For VOPO_4_·2H_2_O, a weight loss of approximately 12.0% is observed below 250 °C, corresponding to the removal of crystalline water. The residual mass of 88.0% is attributed to the anhydrous VOPO_4_ framework. For VOPO_4_-PANI-CH_3_, the total weight loss up to 600 °C is 27.9%, with a residual mass of 72.1%. After subtracting the contribution of crystalline water (9.8%) from the total weight loss, the remaining weight loss of 18.1% is assigned to the decomposition of intercalated PANI-CH_3_. Accordingly, the actual mass fraction of PANI-CH_3_ in the composite is approximately 18.1 wt% [37]. This confirms the successful incorporation and thermal stability of the organic component, which is crucial for maintaining structural integrity during electrochemical cycling, as residual interlayer water can lead to parasitic reactions with the electrolyte and structural instability during prolonged cycling [37].

**Figure 9:**
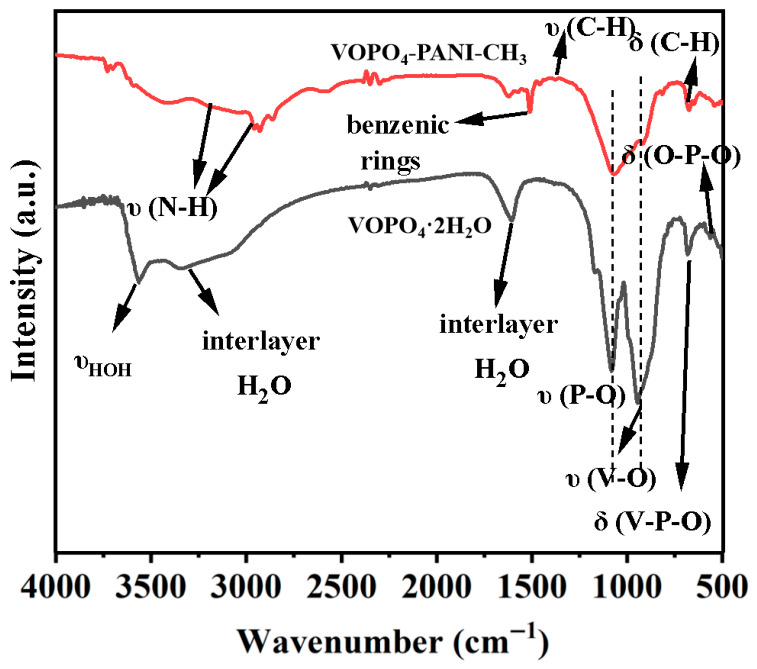
FTIR spectra of VOPO_4_·2H_2_O and VOPO_4_-PANI-CH_3_ hybrids.

FT-IR spectroscopy confirms the successful intercalation of PANI-CH_3_ molecules within the expanded VOPO_4_ host (Figure 9). The pristine VOPO_4_·2H_2_O displays characteristic bands at around 1138, 951, 672 and 556 cm^−1^, which are attributed to υ(P-O), υ(V-O), δ(V-P-O), and δ(O-P-O) vibration modes. The vibrational bands located at 3300 and 1620 cm^−1^ are probably associated with the interlayer water in the host [45]. The band at about 3580 cm^−1^ can be assigned to the bending vibrational mode of water molecules bounded to vanadium site υ(HOH). Upon intercalation, the VOPO_4_-PANI-CH_3_ composite exhibits new characteristic peaks at 3420/3205, 1005, 1340 and 765 cm^−1^, which are indexed to υ(N-H), benzenic rings, υ(C-H), and δ(C-H) bonds of PANI-CH_3_, respectively. It is worth noting that the υ(HOH) stretching vibration absorption and interlayer water-related bands of the VOPO_4_-PANI-CH_3_ electrode appear to weaken or disappear compared to VOPO_4_·2H_2_O, which indicate the substitution of interlayer water molecules caused by the intercalated PANI-CH_3_ species. This expanded interlayer spacing, free of water molecules, facilitates rapid K^+^ diffusion and accommodates volume changes, directly contributing to the enhanced rate capability and cycling stability discussed earlier.

**Figure 10:**
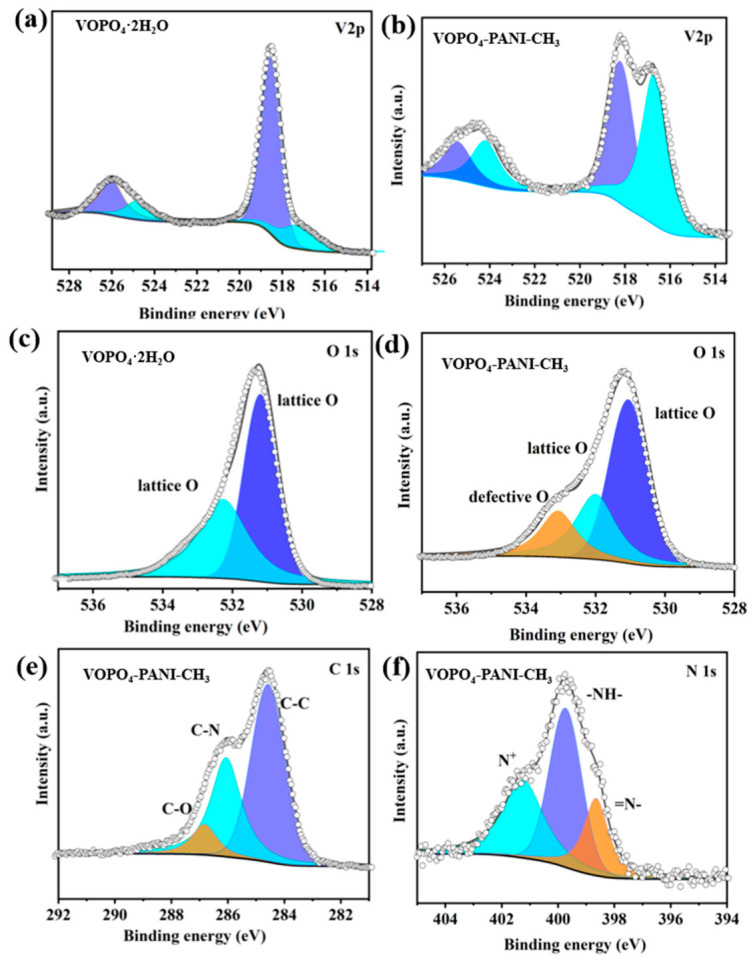
High-resolution XPS spectra of (**a**) V 2p for VOPO_4_·2H_2_O hybrids, (**b**) V 2p for VOPO_4_-PANI-CH_3_ hybrids, (**c**) O 1s for VOPO_4_·2H_2_O hybrids, (**d**) O 1s, (**e**) C 1s, and (**f**) N 1s for VOPO_4_-PANI-CH_3_ hybrids.

The XPS analysis provides further insight into the chemical environment and oxidation states of VOPO_4_-PANI-CH_3_, as shown in Figure 10. In the V 2p^3/2^ spectrum of the VOPO_4_·2H_2_O sample (Figure 10a), two distinct peaks located at 517.5 and 516.3 eV are related to V^5+^ and V^4+^, respectively. In contrast, the VOPO_4_-PANI-CH_3_ composite exhibits a higher V^4+^ proportion compared to pristine VOPO_4_·2H_2_O, suggesting a partial reduction in the vanadium oxide framework upon PANI-CH_3_ intercalation (Figure 10b). This reduction is likely facilitated by electron transfer from the electron-rich phenylamine derivative to the V^5+^ centers, accompanied by the formation of oxygen vacancies within the [VO_n_] layers to maintain charge neutrality [46,47]. Compared with the pristine VOPO_4_·2H_2_O (Figure 10c), the O 1s spectrum of the VOPO_4_-PANI-CH_3_ composite deconvoluted into three typical peaks: the lattice oxygen peak at 531.0 eV, interlayer water peak ≈ 533.1 eV, and an enhanced peak at 532.1 eV assignable to defective oxygen species (Figure 10d) [48]. The generated oxygen vacancies can enhance the electronic conductivity of the electrode material by introducing donor states near the conduction band, and provide additional active sites for K^+^ storage through a defect-mediated adsorption mechanism. The higher V^4+^ content observed in VOPO_4_-PANI-CH_3_ may therefore contribute to its superior specific capacity, complementing the intercalation capacity of the layered host. In the high-resolution C 1s spectra, it is deconvoluted into peaks corresponding to C=C (284.8 eV), C–N (286.2 eV), and –C–O (286.8 eV) bonds, further confirming the presence of PANI-CH_3_ (Figure 10e) [49]. The deconvoluted N 1s spectrum (Figure 10f) displays three distinct peaks at binding energies of 388.5, 389.8, and 401.4 eV, corresponding to the quinoid imine (=N-), benzenoid amine (-NH-), and positively charged nitrogen species (N^+^), respectively, characteristic of the different nitrogen environments in the PANI-CH_3_ molecule backbone. The presence of N^+^ species indicates partial oxidation of the polyaniline derivative, which may participate in charge compensation during electrochemical cycling through reversible redox reactions of the conducting polymer. This additional redox activity could contribute to the overall capacity, particularly at high rates where surface-confined processes dominate.

**Figure 11:**
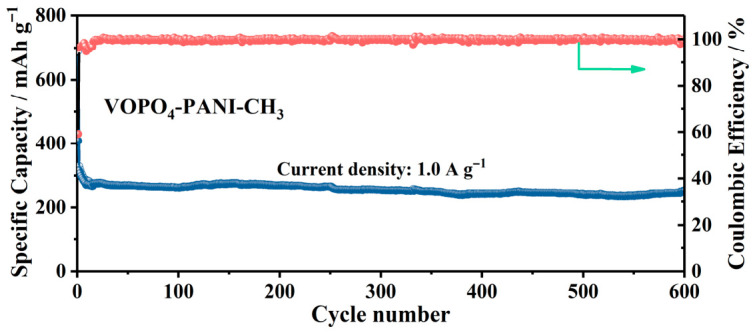
Long-term cycling performance of the VOPO_4_-PANI-CH_3_ electrode at 1 A g^−1^.

**Figure 12:**
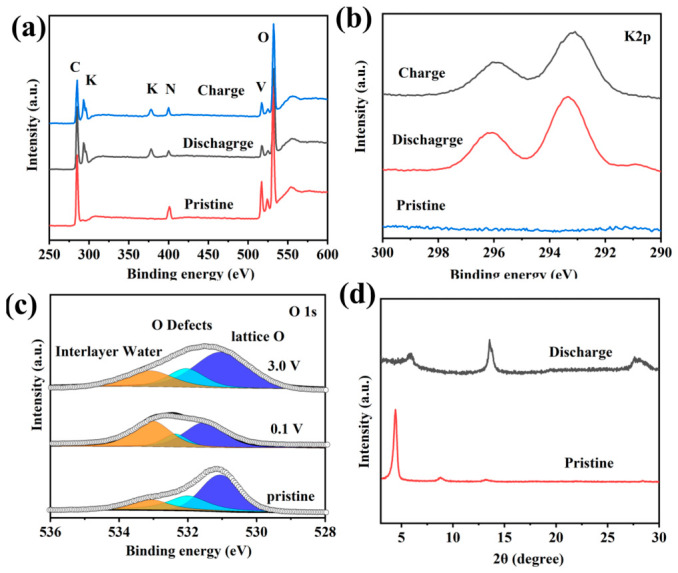
XPS spectra of (**a**) full spectrum, (**b**) K 2p and (**c**) O 1s for the VOPO_4_-PANI-CH_3_ electrode and cycled electrode at fully charged/discharged state. (**d**) XRD patterns of the VOPO_4_-PANI-CH_3_ electrode at fully charged state after 50 cycles.

To further evaluate the long-term electrochemical stability of the VOPO4-PANI-CH_3_ electrode, prolonged cycling was performed at a high current density of 1.0 A g^−1^. As displayed in Figure 11, the VOPO_4_-PANI-CH_3_ electrode delivers a stable reversible capacity of 205.7 mAh g^−1^ over 600 cycles, with the Coulombic efficiency rapidly approaching and subsequently stabilizing at about 100% throughout the test. This excellent cycling stability of VOPO_4_-PANI-CH_3_, combined with the superior rate capability demonstrated earlier, underscores the effectiveness of the PANI-CH_3_ intercalation strategy. The intercalated PANI-CH_3_ serves a dual function: it not only expands the interlayer spacing for VOPO_4_ to facilitate rapid K+ diffusion, but also acts as a structural scaffold that buffers volume variations and maintains electrode integrity during repeated K+ insertion/extraction [50]. To probe the complexity of intercalated species in the KFSI electrolyte, we analyzed the chemical nature of the VOPO_4_-PANI-CH_3_ electrode during different charge/discharge states using XPS. Stable interlayer incorporation of PANI-CH_3_ is evidenced by the persistent N signal (Figure 12a). In contrast to the fresh electrode, the appearance of K 2p peaks upon discharging to 0.1 V, followed by their decline upon charging to 3.0 V (Figure 12b), reveals reversible K^+^ insertion/extraction or the adsorbed species on the electrode surface. In particular, the signals of O 1s show an increase in interlayer water and a decrease in oxygen defects during discharge states, indicative of lattice water migration to the surface and the dynamic role of oxygen vacancies. Upon charging back to 3.0 V, the peak intensity concurrently decreases again, implying that the defect level recovers (Figure 12c) [51]. The phase evolution of the VOPO_4_-PANI-CH_3_ electrode during charge/discharge states was also investigated via XRD analysis (Figure 12d). The (001) peak shifts toward higher angles at full discharge, implying that slight interlayer contraction is caused by K^+^ interaction (Figure 12d), thereby confirming good structural reversibility of the expanded layered structure. Similar phenomena have been exhibited in VOPO_4_-based electrode materials [44]. Consequently, the synergistic effect of expanded interlayer galleries and enhanced structural robustness endows the VOPO_4_-PANI-CH_3_ composite with optimized potassium-ion storage performance.

**Figure 13:**
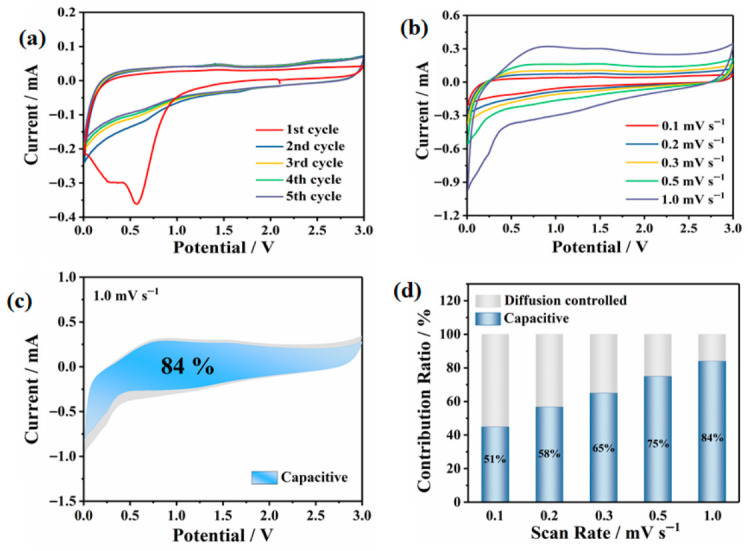
CV curves of VOPO_4_-PANI-CH_3_ at 0.1 mV s^−1^ (**a**) and at different scan rates (**b**), respectively. (**c**) Capacitive contribution at 1.0 mV s^−1^. (**d**) Contribution ratio for VOPO4-PANI-CH3 electrodes regarding capacitive and diffusion contribution at various scan rates, respectively.

The high reversibility of K^+^ intercalation/deintercalation processes in the VOPO_4_-PANI-CH_3_ electrode is evidenced by the symmetric redox peaks observed in the cyclic voltammogram (CV) profiles at a scan rate of 0.1 mVs^−1^ within the voltage range of 0.01–3 V (Figure 13a). Furthermore, the well-defined symmetry of these redox peaks in the CV curves suggests a stepwise (multiphase) potassium-ion storage mechanism of K^+^. To gain deeper insight into the potassium-ion storage kinetics of the VOPO_4_-PANI-CH_3_ electrode, pseudocapacitive analysis was conducted by analyzing the CV curves at various scan rates from 0.2 to 1.0 mVs^−1^ (Figure 13b). Upon the scan rate increasing, the reduction peaks shift toward lower potentials, while the oxidation peaks move toward higher potentials, indicating increased polarization at higher rates. It is essential to distinguish two main potassium storage mechanisms: diffusion-controlled intercalation behaviors and surface-induced capacitive processes, respectively. The respective contributions of the two mechanisms were quantitatively evaluated by the equation $i\left(v\right)=k_{1}v+k_{2}v^{1/2}$, where $k_{1}v$ and $k_{2}v^{1/2}$ respectively correspond to the current contributions from capacitive processes and diffusion-controlled intercalation reactions. A detailed analysis of the capacitive contribution of the VOPO_4_-PANI-CH_3_ electrode at a scan rate of 1.0 mV s^−1^ was determined to be as high as 84%, as illustrated by the shaded region in Figure 13c. Meanwhile, with an increasing scan rate from 0.1 to 0.9 mV s^−1^, the pseudocapacitive contribution of the VOPO_4_-PANI-CH_3_ electrode exhibits a progressive increase from 40.0 to 84.6% (Figure 13d). This pronounced pseudocapacitive process facilitates superior charge storage and rapid reaction kinetics in KIBs, thereby leading to high rate performance and robust cycling stability demonstrated by the VOPO_4_-PANI-CH_3_ electrode [52].

Based on the comprehensive structural, chemical, and electrochemical analyses presented above, a clear structure-performance correlation can be established for the VOPO4-PANI-CH_3_ composite. The expanded interlayer spacing (1.85 nm) provides enlarged diffusion pathways that facilitate rapid K_+_ transport. Simultaneously, the reinforced organic-inorganic interfaces, achieved through the replacement of weakly bonded water molecules by strongly interacting PANI-CH_3_, enhance the structural stability of the host framework, buffering volume variations during repeated K+ insertion/extraction and preserving electrode integrity upon prolonged cycling. The partial reduction in V^5+^ to V^4+^ upon intercalation introduces oxygen vacancies that further enhance electronic conductivity and provide additional active sites for potassium storage. This synergistic combination of expanded interlayer galleries, stabilized interfaces, and defect-engineered electronic structure enables the VOPO_4_-PANI-CH_3_ electrode to simultaneously achieve high reversible capacity, superior rate capability, and exceptional cycling stability. Compared with other recently reported layered materials for potassium-ion batteries, the advanced VOPO_4_-PANI-CH_3_ electrode exhibits a well-balanced performance with high specific capacity and superior cycling durability (Table 1).

## 4. Discussion

In summary, we have successfully demonstrated a rational interlayer engineering strategy to enhance the potassium-ion storage performance of layered VOPO_4_·2H_2_O through the intercalation of a series of polyaniline derivatives with tailored molecular sizes. Depending mainly on the molecular size and substituent groups of the phenylamine derivatives (PA, PTA and PMA), the interlayer spacing was gradually expanded from the pristine value of 0.76 nm (VOPO_4_) to 1.58 nm (VOPO_4_-PANI), 1.85 nm (VOPO_4_-PANI-CH_3_), and 2.09 nm (VOPO_4_-PANI-(CH_2_)_3_CH_3_), respectively. Among the three composites, the VOPO_4_-PANI-CH_3_ electrode with a preferred interlayer spacing of 1.85 nm delivers a high reversible capacity of 333.2 mA g^−1^ over 100 cycles at 0.1 A g^−1^. The expanded interlayer spacing and reinforced organic-inorganic interfaces synergistically improve K^+^ storage performance, accelerating reaction kinetics while maintaining structural integrity upon cycling, thereby enabling superior cycling stability. This work provides molecular-level intercalation engineering by tailored aniline derivatives to design high-performance layered anode materials for next-generation KIBs. The fundamental insights gained into the structure-performance relationship offer valuable guidance for the rational design of advanced energy storage materials.

## Figures and Tables

**Table 1:** Comparison of the electrochemical performance in this work with the recently reported layered KIB electrodes for potassium-ion batteries.

Electrode Materials	Voltage Window (V)	Electrolyte	Specific Capacity (mAh g^−1^ at A g^−1^)	Specific Capacity After Long-Term Cycle (mAh g^−1^ at Ag^−1^)	Reference
K_0.25_TiS_2_ phase	1.0–3.0 V	1.0 M KPF_6_	123.0/0.1	91.0 (1 Ag^−1^ over 2000 cycles)	[4]
1T-WSe_2_-Sn	0.01–3.0 V	3.0 M KFSI	345/0.1	120 (at 1.0 Ag^−1^ for 1000 cycles)	[53]
V_5_Se_8_@C hybrids	0.01–2.6 V	1 M KFSI	327/0.1	145 (4 Ag^−1^ after 800cycles)	[54]
MoS_2_ with expanded interlayer	0.01–3.0 V	1 M KFSI	510/0.2	310 (1 Ag^−1^ over 100 cycles)	[30]
WSe_2_/C layered nanosheets	0.01–2.5 V	1 MKFSI	384/0.1	209 (1 Ag^−1^ after 500 cycles)	[31]
VOPO_4_-PANI-CH_3_ electrode	0.1–3.0 V	1 M KFSI	333.2/0.1	205.7 (1 Ag^−1^ after 600 cycles)	This work

## Data Availability

As these data are also part of an ongoing study, it is not currently possible to share the raw/processed data required to replicate the results of these studies.
